# Modeling the effectiveness of nebulized terbutaline for decompensated chronic obstructive pulmonary disease patients in the emergency department

**DOI:** 10.1097/MD.0000000000004553

**Published:** 2016-08-12

**Authors:** Florian Gueho, Sébastien Beaune, Philippe Devillier, Saik Urien, Christophe Faisy

**Affiliations:** a Department of Emergency Medicine, Groupe Hospitalier Carnelle Portes de L’Oise, Beaumont-Sur-Oise; b Department of Emergency Medicine, Ambroise Paré Hospital, Assistance Publique – Hôpitaux de Paris, Boulogne-Billancourt; c Research Unit UPRES EA220, University Versailles Saint–Quentin, Foch Hospital, Suresnes; d Clinical Investigations Center-1419 INSERM, EAU-08 University Paris Descartes Sorbonne Paris Cité, Paris, France.

**Keywords:** chronic obstructive pulmonary disease decompensation, emergency department, pharmacodynamics, short acting β_2_-agonists, terbutaline

## Abstract

Short-acting β_2_-agonists (SABA) are widely used in the emergency department (ED) to treat patients with decompensated chronic obstructive pulmonary disease (COPD). We sought to model the effectiveness of nebulized SABA (terbutaline) on clinically relevant parameters associated with a reduction in work of breathing or respiratory muscle fatigue in decompensated COPD patients admitted to the ED.

Forty consecutive decompensated COPD patients (having received at least one dose of nebulized terbutaline during their stay in the ED) were included in an observational cohort study. The terbutaline dose received at time *t* was expressed as cumulative dose and as a rate (mg/day). The associations between the terbutaline dose and time-dependent outcome parameters (respiratory rate, heart rate, arterial blood gases, and, as a marker of terbutaline's systemic effect, serum potassium) were analyzed using a nonlinear, mixed-effects model. The effect of various covariates influencing terbutaline's effectiveness (baseline characteristics and concomitant treatments) was assessed on the model.

Among the investigated patients, a total of 377 time-dependent observations were available for analysis. Neither the cumulative dose nor the dose rate at time *t* significantly influenced the arterial blood gas parameters or heart rate. The cumulative dose of terbutaline was associated with a lower serum potassium level (*P* < 0.001) and, less significantly, a lower respiratory frequency (*P* = 0.036). In a tertile analysis, the need for post-ED hospitalization was not associated with the cumulative dose or dose rate of terbutaline.

Overall, the results of our modeling study strongly suggest that terbutaline dose did not influence time-dependent outcomes other than serum potassium, and thus call into question the systematic administration of inhaled SABA to patients admitted to the ED for decompensated COPD.

## Abstract 2

Supplemental Digital Content is available in the text

## 1 Introduction

Short-acting β_2_-agonists (SABA) are widely used in the emergency department (ED) to treat patients with decompensated chronic obstructive pulmonary disease (COPD). ^[1]^ The rationale for this treatment is based on the decrease in airway resistance, which (in theory, at least) reduces the work of breathing and thus respiratory muscle fatigue. Surprisingly, SABA have never been tested against clinically relevant criteria (e.g., a reduction in respiratory muscle fatigue or the need for post-ED hospitalization in a normal ward or an intensive care unit (ICU)) in a placebo-controlled clinical trial. ^[1]^ This is all the more surprising in view of the knowledge that anticholinergic drugs produce similar short-term improvements in the forced expiratory volume in one second (FEV_1_) and respiratory symptoms (although their bronchodilatory effect is slower) and could be used instead of SABA. ^[2]^ It is also well known that the use of repeated doses of SABA to treat decompensated COPD or asthma in the ED is associated with adverse events (i.e., changes in PaO_2_, serum glucose, and serum potassium). ^[3]^ Moreover, the use of β_2_-agonists alone might have harmful effects by enhancing nonspecific airway responsiveness and inflammation, which has been observed in vitro.[ ^4^ ^5^] In asthma, small but statistically significant increases in respiratory and asthma-related deaths were observed in a large US study of patients treated with salmeterol. ^[6]^ However, the results of post hoc analyses suggested that this phenomena could be also explained by low socioeconomic status and the cessation of treatment with inhaled corticosteroids. In the context of COPD, there is no evidence of elevated exacerbation or mortality rates in long-term users of long-acting β_2_-agonists (LABA).

The worldwide economic burden of COPD decompensation (including SABA cost) has been estimated at billions of dollars. ^[1]^ In decompensated COPD, the main expected benefits of reducing airway resistance and respiratory muscle fatigue via SABA administration include a decreased need for noninvasive or invasive mechanical ventilation and the reduced admission to ICU or intermediate respiratory care units (therefore reducing morbidity/mortality rates and the economic burden). ^[7]^ The primary objective of the present preliminary study was therefore to assess the effectiveness of a nebulized SABA (terbutaline, which is extensively used in French EDs) on clinically relevant outcomes in patients with decompensated COPD. We subsequently modeled the effectiveness of nebulized terbutaline in the ED on clinically relevant parameters associated with a reduction in work of breathing or in respiratory muscle fatigue.

## 2 Methods

### 2.1 Design and setting

This observational cohort study was performed from January 2012 to June 2013 in the ED (which includes a short-stay unit) of a tertiary teaching hospital in France. In accordance with the French legislation on observational studies, approval by an investigational review board was not required. The study's confidential use of electronically processed patient data was approved by the French National Commission for Data Protection (*Commission Nationale de l’Informatique et des Libertés*; reference: 1922081).

### 2.2 Data sources and patient selection

Data were extracted from the medical records of patients admitted to the ED for decompensated COPD (i.e., a fast, reversible deterioration of the respiratory status due to acute disease, exacerbation of inflammatory phenomena affecting the bronchi or core symptoms such as cough, dyspnea, and bronchorrhea) and who had received at least one dose of nebulized terbutaline. Patients over the age of 40 years and with a history of COPD (according to the 2007 Global Initiative for Chronic Obstructive Lung Disease criteria) were included in the analysis. ^[1]^ The main exclusion criteria were acute asthma or chronic asthma, cystic fibrosis or diffuse bronchiectasis, a history of lobectomy, pneumonectomy, or tracheotomy, noninvasive ventilation at home, known allergy to SABA or a permanent SABA contraindication, decompensated chronic comorbidities, unfeasible home care, missing data in the medical records, or moribund patients (life expectancy < 3 weeks). All data were collected by the same investigator. Two other senior investigators with expertise in respiratory disease then confirmed the eligibility of the collected data for analysis by consensus; this limited the number of confounding factors by excluding patients in whom the diagnosis of decompensated COPD was uncertain.

### 2.3 Terbutaline administration

Nebulized terbutaline (5 mg/2 mL) was administered with a single-use, air-driven nebulizer (gas flow rate: 6 L/min for 15 minutes). If the patient's respiratory status failed to improve, terbutaline was combined with ipratropium (0.5 mg/2 mL). Other treatments were administered according to standard guidelines. ^[8]^ Patients were transferred out of the ED (to the ICU, the medicine department, or home) in accordance with national and international guidelines.[ ^8^ ^9^] Patients were transferred to the ICU if they met any of the following criteria: severe acidosis (pH < 7.30), worsening hypoxia despite oxygen treatment, agitation, or depressed mental status, or the need for respiratory support. Patients were transferred to the medicine department if they displayed worsening baseline hypercapnia (without severe acidosis) or persistent dyspnea. All other patients were discharged to home. For the analyses, terbutaline was expressed as a cumulated dose at time *t*, TOTDOSE _(*t*)_, or as a rate (mg/d) as follows: RATE_(*t*)_ = TOTDOSE_(*t*)_/*t*.

### 2.4 Data collection

The baseline characteristics of the included patients were recorded on admission to the ED. The simplified acute physiology score (SAPS) II and nutrition risk score (NRS) were used to assess the severity of illness and protein-calorie malnutrition, respectively.[ ^10^ ^11^] Data on arterial blood gases, the respiratory rate, the heart rate, and the serum potassium level (a marker of the systemic effect of terbutaline) available before and up to 4 hours after nebulization of terbutaline were collected from the patient's records. We also recorded covariates known to potentially influence the pharmacodynamics of terbutaline: the reversibility of airway obstruction in lung function tests, chronic left ventricular failure, a history of glucocorticoid or bronchodilator use, and concomitant treatments with glucocorticoids, other bronchodilators, or drugs interacting with the airway smooth muscle tone (calcium channel blockers, beta blockers, or nonsteroidal anti-inflammatory drugs). ^[12]^

### 2.5 Outcome measures

The main outcome criteria were the partial carbon dioxide pressure in arterial blood (PaCO_2_), the respiratory rate, and the need for post-ED hospitalization (whatever the destination). Secondary outcomes included heart rate, oxygen pressure in arterial blood (PaO_2_), arterial pH, serum bicarbonates, and serum potassium.

### 2.6 Data analysis and modeling

The R statistical software (https://www.r-project.org) was used for all statistical analyses. Time-dependent outcomes (repeated measures) were analyzed using a linear mixed-effects model with the lme4 package (https://cran.r-project.org/web/packages/lme4/index.html). The sample size was estimated a priori, based on the general rule whereby 10 events of the outcome of interest are required for each variable in the model that includes the exposure of interest. Univariate analyses were first conducted to evaluate the contribution of specific factors on the different outcomes (Table S1, see supplemental digital content). Variables with *P* < 0.20 were retained for multivariate analyses. Thereafter, the covariates were removed or retained in the model on the basis of the Akaike Information Criterion. The final multivariate model systematically included terbutaline plus those retained covariates. Missing data were only imputed in the models for respiratory rate. The effects of terbutaline and covariates on outcome variables were ascribed to the following equation/formula: Effect = *b*_T_ × TERBUTALINE (*t*) + *b*_C_ × COV + (1|subject) + ε, where *b*_T_ and *b*_C_ are typical slope coefficients for TERBUTALINE (*t*) and other variables (COV) effects; (1|subject) denotes the intercept that is different for each subject; ε is a general error term accounting for the random differences between different scenarios from the same subject. A positive slope means that the variable and the outcome increase together, whereas a negative slope means that the outcome decreases as the variable increases. The threshold for statistical significance was set to *P* < 0.05. Relationships between the tertiles of the total cumulated dose (TCD) at the end of the ED stay or of the mean dose rate, MDR (MDR = TCD/ED_time_), and the need for post-ED hospitalization were assessed by a χ^2^ test for trend in proportions.

## 3 Results

### 3.1 Characteristics of the study population, and time-dependent observations

**Figure 1:**
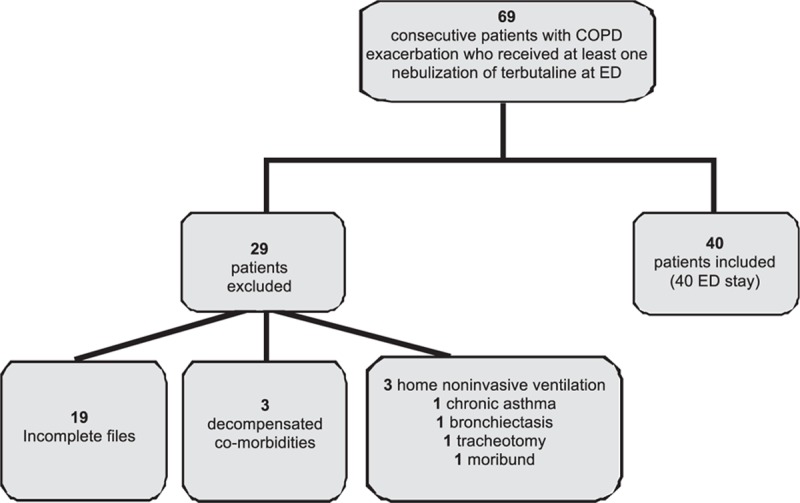
Study flow chart. COPD = chronic pulmonary disease, ED = emergency department.

During the study period, 69 consecutive patients with decompensated COPD and having received at least one dose of nebulized terbutaline in the ED were eligible for analysis. Twenty-nine patients met the exclusion criteria, so a total of 40 patients were included in the modeling analysis of terbutaline's effectiveness (Fig. 1). The clinical characteristics of the study population are summarized in Table 1. The most frequent concomitant treatment associated with terbutaline in the ED was ipratropium (Table 1). For the 40 investigated patients, a total of 377 time-dependent observations (170 respiratory rate, 79 heart rate, 67 arterial blood gases, and 61 serum potassium) were available for analysis. The median (range) values were as follows: respiratory rate: 28 (17–40) cycles/min; heart rate: 88 (50–142) beats/min; PaCO_2_: 45 (29–120) mm Hg; serum bicarbonates: 28 (20–38) mmol/L; pH: 7.40 (7.09–7.51); PaO_2_: 64 (43–111) mm Hg; serum potassium: 4.2 (2.8−6.2) mmol/L.

**Table 1:**
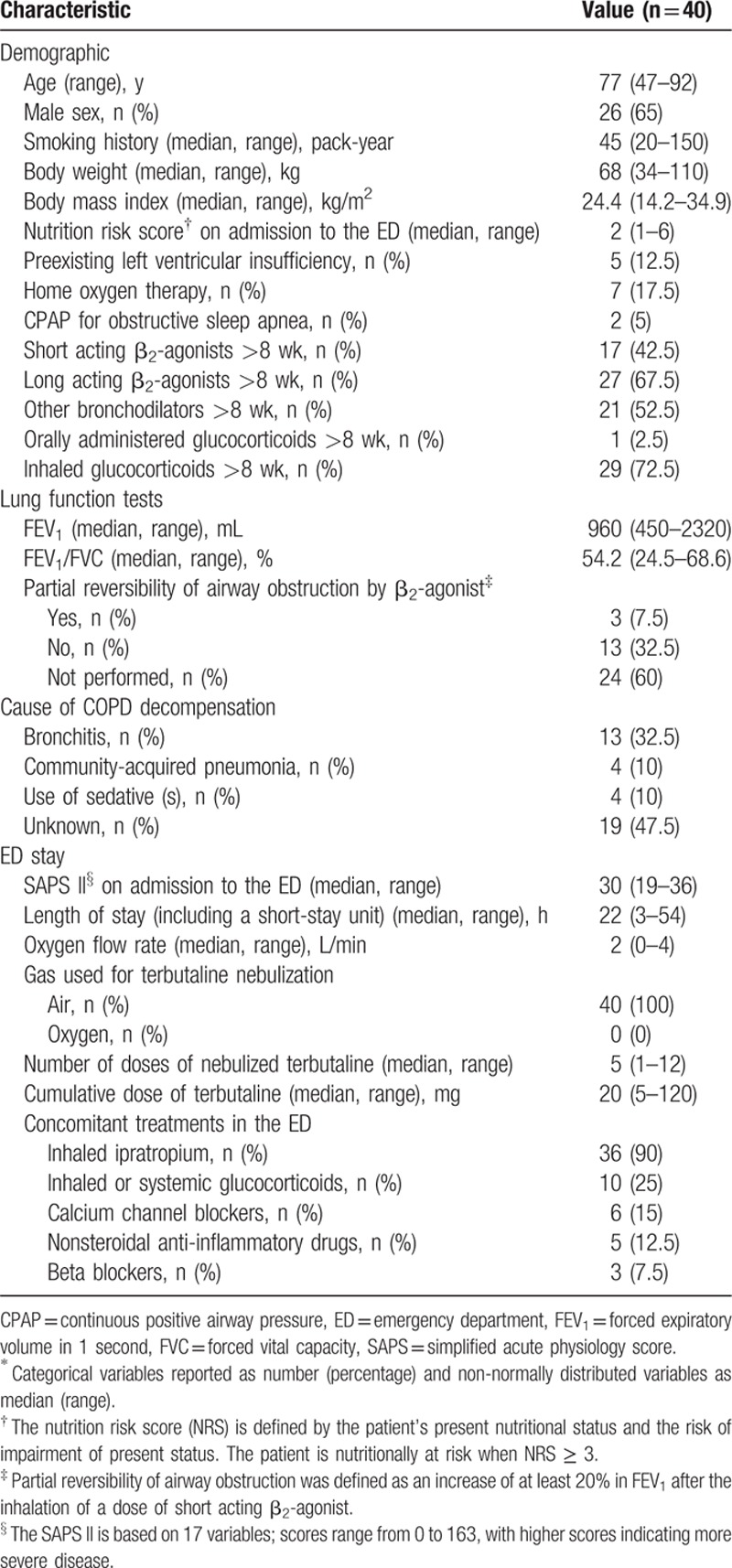
Baseline characteristics of the study population and management in the ED^∗^.

### 3.2 Effect of terbutaline dosage on time-dependent outcomes in the ED

Neither the cumulative dose of terbutaline nor the dose rate at time *t* significantly influenced the arterial blood gas parameters or heart rate (Table 2). The cumulative dose of terbutaline at time *t* was associated with lower serum potassium levels and respiratory rate. Dose rate at time *t* showed similar but not significant trend for all outcomes (Table 2).

**Table 2:**
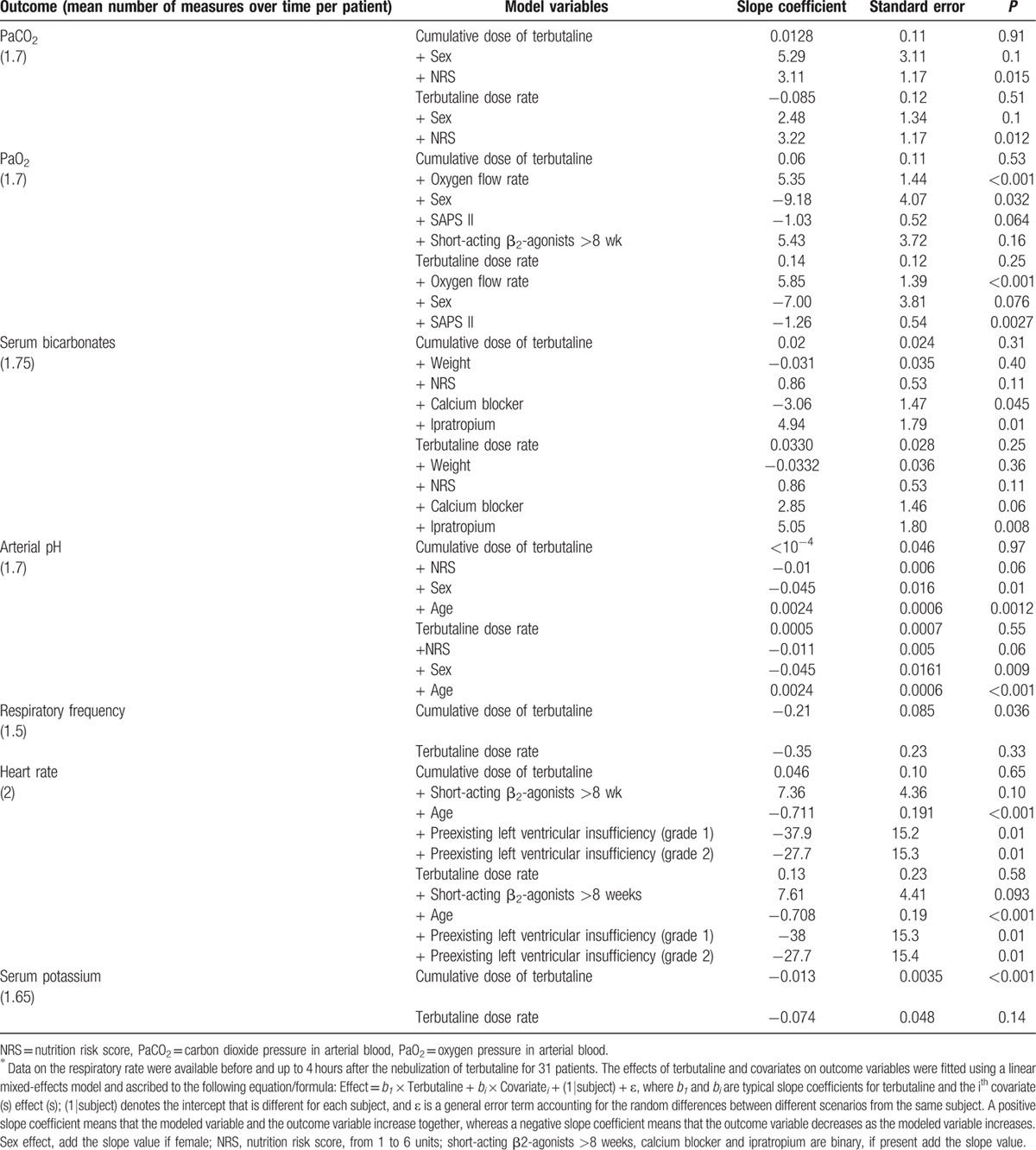
Influence of terbutaline and covariates on time-dependent outcome variables in 40 patients with decompensated COPD^∗^.

### 3.3 Influence of the terbutaline dose on the need for post-ED hospitalization

**Figure 2:**
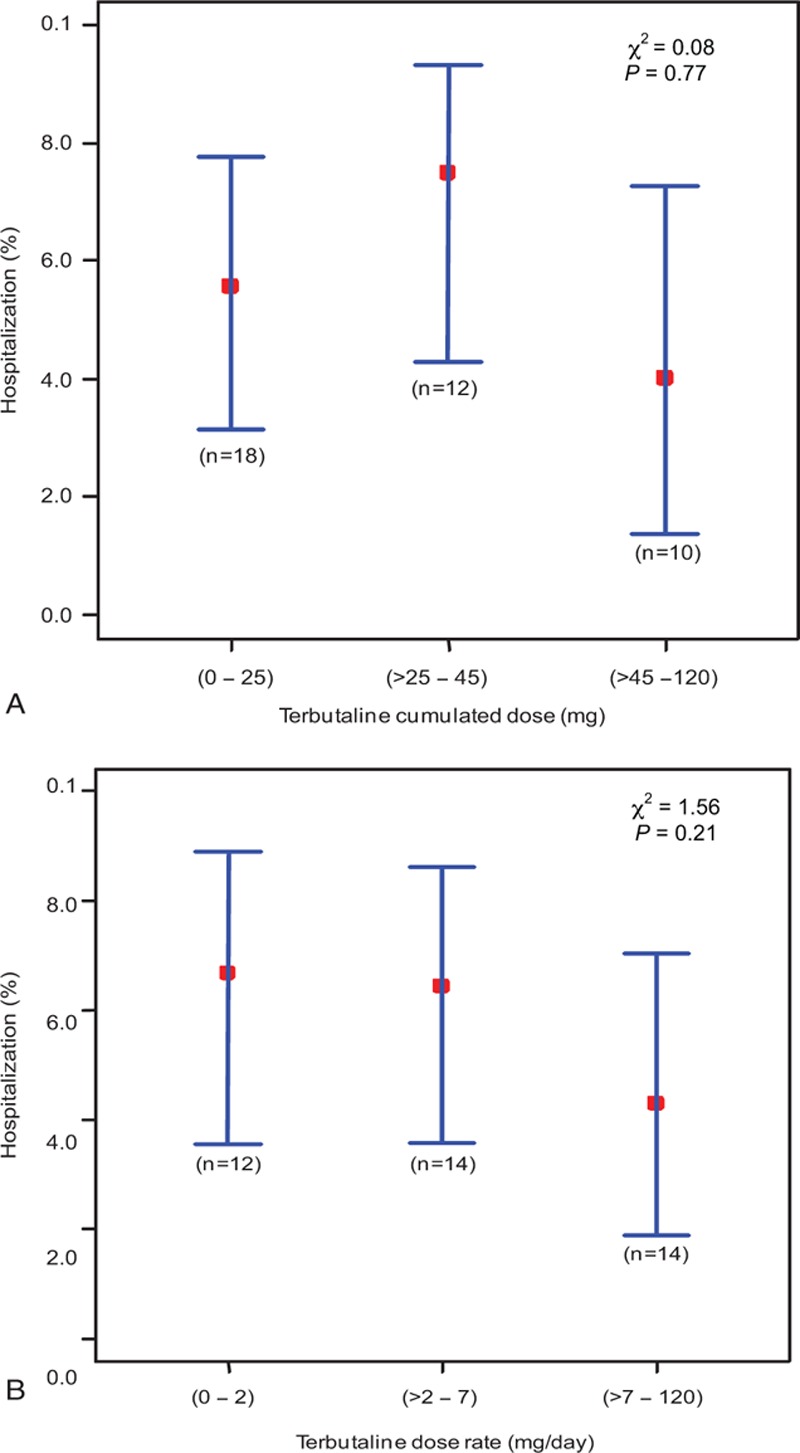
Relationship between the need for hospitalization and the tertiles of the cumulative dose of terbutaline (A) or the dose rate (B) during a stay in the ED for 40 patients with decompensated COPD. Data are presented as percentages (red squares), with the corresponding 95% confidence interval (vertical blue lines). In a Mantel–Haenszel test, there were no significant intertertiles differences.

Thirty patients (75%) were transferred to the medicine department, 8 (20%) were discharged to home, and 2 (5%) were admitted to the ICU. No significant trend for hospitalization across the cumulative dose of terbutaline or dose rate tertiles was found (Fig. 2).

## 4 Discussion

In this modeling analysis, the cumulative dose and dose rate of terbutaline were not associated with significant differences in clinically relevant parameters associated with a reduction in work of breathing or in respiratory muscle fatigue in patients admitted to the ED for COPD decompensation. There was only a weak respiratory rate decrease, minus 2/min for each additional 10 mg terbutaline dose, with *P* value at the limit of significance (*P* < 0.05). The reduction in work of breathing by β_2_-agonists has been generally observed in mechanically ventilated patients but not in spontaneously breathing patients such as COPD patients in the ED. ^[13]^ Moreover, inhaled terbutaline was seen to have a systemic effect since it was associated with a dose-dependent decrease in the serum potassium level. These preliminary results challenge assumptions concerning the effectiveness/safety ratio of inhaled SABA, and emphasize the need to assess the benefit of SABA in a large, controlled clinical trial in this context.

Several factors may have contributed to the absence of clinically relevant terbutaline respiratory effects. Few patients presented with proven, reversible airway obstruction (using a β_2_-agonist) in lung function tests. Most patients had received β_2_-agonists or other bronchodilators in the weeks preceding admission to the ED; associated β_2_-adrenoceptor saturation, desensitization, or downregulation in airway smooth muscle may have limited the effectiveness of inhaled SABA.[ ^14^ ^15^] However, chronic use of salmeterol does not compromise the bronchodilatory response to albuterol during acute episodes of asthma. ^[16]^ Proinflammatory cytokines such as IL-1β and TNF-α can synergize to promote the in vitro β-adrenergic hyporesponsiveness of human airway smooth muscle and thus may contribute to a weak bronchodilatory response during COPD decompensation. ^[17]^ Moreover, unfavorable mechanical properties of the respiratory tract can be observed in severely flow-limited COPD patients and may explain why the bronchodilator effect of terbutaline did not appear to produce a reduction in PaCO_2_ by decreasing work of breathing. Bronchial edema during COPD decompensation may also reduce the beneficial effect of terbutaline-induced airway smooth muscle relaxation. The response to treatment with β_2_-agonists is heterogeneous in individuals with asthma or COPD; this might be due (at least in part) to genetic variation in the β_2_-adrenergic receptor gene.[ ^14^ ^18^] In fact, β_2_-adrenoceptor gene polymorphism is unlikely to account for our present results; in contrast to the situation in cardiac diseases, responses to β_2_-agonists in respiratory diseases slightly vary with receptor polymorphism.[ ^19^ ^20^ ^21^] However, the present study was not designed to answer this question.

Depending on the cellular context and a specific ligand's intrinsic activity, β_2_-adrenoceptor stimulation can couple with G_i_ heterotrimeric proteins (instead of G_s_ proteins) or activate proinflammatory, intracellular signaling pathways via β-arrestin (a G protein-coupled receptor kinase). ^[22]^ Specific conformational changes at key sites (9 cysteine and lysine residues) on the β_2_-adrenoceptor are involved in these changes in functional selectivity and biased agonism. ^[23]^ Furthermore, pharmacological investigations have shown that intracellular proinflammatory pathways are activated by prolonged exposition (>15 hours) to β_2_-agonists; this leads to the sensitization of airway smooth muscle, airway neuroinflammation, and dysfunction of the epithelial regulation of airway smooth muscle contraction.[ ^4^ ^5^] These untoward effects may counterbalance the relaxant effect of SABA in COPD patients. ^[12]^ Moreover, a protective effect of corticosteroids is less frequent in COPD patients than in asthmatics, in whom inhaled corticoid therapy is more common. In the present study, almost three quarters of the COPD patients had undergone long-term inhaled corticosteroid therapy before admission to ED, whereas only a quarter received inhaled or orally administered glucocorticoids during their stay in the ED.

In COPD patients, long-term treatment with β_2_-agonists has proven its effectiveness in terms of reduced respiratory symptoms and (to a lesser extent) higher FEV_1_ values. This is why SABA are part of the standard of care for patients with decompensated COPD despite the fact that the approach's efficacy has never been established in a randomized trial. ^[1]^ However, some researchers consider that a placebo-controlled study of the efficacy of SABA for COPD decompensation in the ED would be unethical. Our present results question this position.

One limitation of this preliminary study relates to the relatively small number of time-dependent observations from a single center; thus, caution should be applied before extrapolating the results to other patient populations and other ED. Although a larger sample size might have revealed a statistically significant effect, the effect size is undoubtedly small and thus would be of questionable clinical significance. Data were collected retrospectively from records filled out by the ED staff, which may thereby have introduced bias into the study. Nevertheless, the study's retrospective, observational design limited the Hawthorne effect and may provide a more realistic picture of “real-life” practice in which the standardized management of COPD exacerbation in French EDs was based on international guidelines.[ ^1^ ^8^ ^9^] Another limitation relates to the possible diagnostic overlap between COPD exacerbation and other acute respiratory comorbidities (such as pneumonia or the use of sedatives), ^[1]^ which would have increased the heterogeneity of the study population. However, our exclusion of decompensated chronic comorbidities increased the homogeneity of the study population and limited the number of confounding factors affecting the effectiveness of terbutaline.

Lastly, our results identified only few covariates that influenced terbutaline pharmacodynamics in patients with decompensated COPD in the ED. The only time-dependent outcome significantly influenced by terbutaline was serum potassium. The level of evidence for the systematic use of SABA in decompensated COPD patients admitted to the ED remains low. In conclusion, our preliminary findings suggest that a placebo-controlled study of the systematic use of inhaled SABAs in this context is now warranted.

## Supplementary Material

Supplemental Digital Content
