# Supplementary material for: Modeling the effectiveness of nebulized terbutaline for decompensated chronic obstructive pulmonary disease patients in the emergency department
Source: Medicine (Baltimore). 2016 Aug 12;95(32):e4553. doi: 10.1097/MD.0000000000004553 (PMC4985335; doi:10.1097/MD.0000000000004553)
Supplement: Supplemental Digital Content [file medi-95-e4553-s001.docx]

**Table S1: Univariate analyses for dependent variables (outcomes)**

Models with variable fixed effect (H1) and without variable fixed effect (H0) were fitted using the lmer function of the lme4 R package. The asymptotic likelihood ratio test was achieved by an ANOVA test, anova (H1,H0, test="Chisq"), to estimate the p value for the variable significance.

| **Outcome** | **Variable** | **Chi² statistics** | **p value** |
| --- | --- | --- | --- |
| Arterial pH | Cumulative dose of terbutaline | 0.00 | 1.00 |
| Arterial pH | Terbutaline dose rate | 0.10 | 0.75 |
| Arterial pH | Oxygen flow rate | 0.52 | 0.47 |
| Arterial pH | Age | 4.44 | 0.04 |
| Arterial pH | Bodyweight | 0.03 | 0.86 |
| Arterial pH | SAPS II | 0.44 | 0.51 |
| Arterial pH | NRS | 2.64 | 0.10 |
| Arterial pH | Gender | 2.93 | 0.09 |
| Arterial pH | Pre-existing left ventricular insufficiency | 5.44 | 0.07 |
| Arterial pH | Active smoking | 2.90 | 0.09 |
| Arterial pH | Inhaled glucocorticoids >8 weeks | 6.93 | 0.01 |
| Arterial pH | Orally administered glucocorticoids >8 weeks | 0.55 | 0.46 |
| Arterial pH | Long acting 2-agonists >8 weeks | 5.57 | 0.02 |
| Arterial pH | Short acting 2-agonists >8 weeks | 2.74 | 0.10 |
| Arterial pH | Ipratropium | 0.02 | 0.88 |
| Arterial pH | Calcium blocker | 0.35 | 0.55 |
| PaO2 | Cumulative dose of terbutaline | 1.86 | 0.17 |
| PaO2 | Terbutaline dose rate | 0.85 | 0.36 |
| PaO2 | Oxygen flow rate | 8.69 | 0.00 |
| PaO2 | Age | 1.09 | 0.30 |
| PaO2 | Bodyweight | 1.03 | 0.31 |
| PaO2 | SAPS II | 7.25 | 0.01 |
| PaO2 | NRS | 0.02 | 0.90 |
| PaO2 | Gender | 1.92 | 0.17 |
| PaO2 | Pre-existing left ventricular insufficiency | 0.85 | 0.66 |
| PaO2 | Active smoking | 0.08 | 0.78 |
| PaO2 | Inhaled glucocorticoids >8 weeks | 0.46 | 0.50 |
| PaO2 | Orally administered glucocorticoids >8 weeks | 0.18 | 0.68 |
| PaO2 | Long acting 2-agonists >8 weeks | 0.44 | 0.51 |
| PaO2 | Short acting 2-agonists >8 weeks | 1.84 | 0.18 |
| PaO2 | Ipratropium | 0.04 | 0.84 |
| PaO2 | Calcium blocker | 0.84 | 0.36 |
| PaCO2 | Cumulative dose of terbutaline | 0.00 | 0.96 |
| PaCO2 | Terbutaline dose rate | 0.42 | 0.52 |
| PaCO2 | Oxygen flow rate | 2.81 | 0.09 |
| PaCO2 | Age | 0.16 | 0.69 |
| PaCO2 | Bodyweight | 1.88 | 0.17 |
| PaCO2 | SAPS II | 0.47 | 0.50 |
| PaCO2 | NRS | 10.30 | 0.00 |
| PaCO2 | Gender | 6.43 | 0.01 |
| PaCO2 | Pre-existing left ventricular insufficiency | 2.65 | 0.27 |
| PaCO2 | Active smoking | 0.95 | 0.33 |
| PaCO2 | Inhaled glucocorticoids >8 weeks | 0.05 | 0.83 |
| PaCO2 | Orally administered glucocorticoids >8 weeks | 3.10 | 0.08 |
| PaCO2 | Long acting 2-agonists >8 weeks | 0.42 | 0.52 |
| PaCO2 | Short acting 2-agonists >8 weeks | 1.66 | 0.20 |
| PaCO2 | Ipratropium | 1.38 | 0.24 |
| PaCO2 | Calcium blocker | 1.54 | 0.21 |
| Serum bicarbonates | Cumulative dose of terbutaline | 0.70 | 0.40 |
| Serum bicarbonates | Terbutaline dose rate | 1.11 | 0.29 |
| Serum bicarbonates | Oxygen flow rate | 2.00 | 0.16 |
| Serum bicarbonates | Age | 1.25 | 0.26 |
| Serum bicarbonates | Bodyweight | 2.54 | 0.11 |
| Serum bicarbonates | SAPS II | 2.67 | 0.10 |
| Serum bicarbonates | NRS | 3.75 | 0.05 |
| Serum bicarbonates | Gender | 4.78 | 0.03 |
| Serum bicarbonates | Pre-existing left ventricular insufficiency | 2.46 | 0.29 |
| Serum bicarbonates | Active smoking | 0.01 | 0.93 |
| Serum bicarbonates | Inhaled glucocorticoids >8 weeks | 3.15 | 0.08 |
| Serum bicarbonates | Orally administered glucocorticoids >8 weeks | 4.00 | 0.05 |
| Serum bicarbonates | Long acting 2-agonists >8 weeks | 1.68 | 0.20 |
| Serum bicarbonates | Short acting 2-agonists >8 weeks | 0.23 | 0.63 |
| Serum bicarbonates | Ipratropium | 3.47 | 0.06 |
| Serum bicarbonates | Calcium blocker | 2.27 | 0.13 |
| Heart rate | Cumulative dose of terbutaline | 0.01 | 0.93 |
| Heart rate | Terbutaline dose rate | 0.00 | 0.95 |
| Heart rate | Oxygen flow rate | 0.46 | 0.50 |
| Heart rate | Age | 8.83 | 0.00 |
| Heart rate | Bodyweight | 0.02 | 0.88 |
| Heart rate | SAPS II | 3.13 | 0.08 |
| Heart rate | NRS | 0.30 | 0.58 |
| Heart rate | Gender | 0.59 | 0.44 |
| Heart rate | Pre-existing left ventricular insufficiency | 7.17 | 0.03 |
| Heart rate | Active smoking | 2.28 | 0.13 |
| Heart rate | Inhaled glucocorticoids >8 weeks | 4.01 | 0.05 |
| Heart rate | Orally administered glucocorticoids >8 weeks | 0.35 | 0.55 |
| Heart rate | Long acting 2-agonists >8 weeks | 2.54 | 0.11 |
| Heart rate | Short acting 2-agonists >8 weeks | 1.81 | 0.18 |
| Heart rate | Ipratropium | 0.09 | 0.77 |
| Heart rate | Calcium blocker | 1.29 | 0.26 |
| Serum potassium | Cumulative dose of terbutaline | 12.40 | 0.00 |
| Serum potassium | Terbutaline dose rate | 2.36 | 0.13 |
| Serum potassium | Oxygen flow rate | 3.23 | 0.07 |
| Serum potassium | Age | 4.29 | 0.04 |
| Serum potassium | Bodyweight | 1.25 | 0.26 |
| Serum potassium | SAPS II | 2.49 | 0.11 |
| Serum potassium | NRS | 1.85 | 0.17 |
| Serum potassium | Gender | 0.54 | 0.46 |
| Serum potassium | Pre-existing left ventricular insufficiency | 1.04 | 0.60 |
| Serum potassium | Active smoking | 1.25 | 0.26 |
| Serum potassium | Inhaled glucocorticoids >8 weeks | 0.05 | 0.82 |
| Serum potassium | Orally administered glucocorticoids >8 weeks | 0.28 | 0.60 |
| Serum potassium | Long acting 2-agonists >8 weeks | 0.68 | 0.41 |
| Serum potassium | Short acting 2-agonists >8 weeks | 0.00 | 0.96 |
| Serum potassium | Ipratropium | 0.13 | 0.72 |
| Serum potassium | Calcium blocker | 0.12 | 0.73 |
| Respiratory rate | Cumulative dose of terbutaline | 5.99 | 0.01 |
| Respiratory rate | Terbutaline dose rate | 2.20 | 0.14 |
| Respiratory rate | Oxygen flow rate | 0.04 | 0.84 |
| Respiratory rate | Age | 3.11 | 0.08 |
| Respiratory rate | Bodyweight | 4.55 | 0.03 |
| Respiratory rate | SAPS II | 4.38 | 0.04 |
| Respiratory rate | NRS | 0.20 | 0.65 |
| Respiratory rate | Gender | 0.04 | 0.84 |
| Respiratory rate | Pre-existing left ventricular insufficiency | 0.03 | 0.87 |
| Respiratory rate | Active smoking | 9.51 | 0.00 |
| Respiratory rate | Inhaled glucocorticoids >8 weeks | 2.40 | 0.12 |
| Respiratory rate | Orally administered glucocorticoids >8 weeks | 0.87 | 0.35 |
| Respiratory rate | Long acting 2-agonists >8 weeks | 0.25 | 0.62 |
| Respiratory rate | Short acting 2-agonists >8 weeks | 0.00 | 0.96 |
| Respiratory rate | Ipratropium | 0.16 | 0.69 |
| Respiratory rate | Calcium blocker | 1.10 | 0.30 |
